# The M. D.’s

**Published:** 1884-05

**Authors:** 


### The M. D's.

The *Chicago Medical Journal* says there are on record in the office of the Illinois State Board of Health the written-proof evidences that not less than two hundred graduates of medicine cannot spell correctly the word diploma. The following are seven different methods actually employed: diaploma, diplomy diplomer, diplomah, diaplemy, diapluma, dipluma. Two hundred men in one State having a diploma, when they are unable to spell even the word diploma. Lo, the rottenness in the profession! How can we expect cultured people to rank us among the learned professions when we have so large an ignorant membership?—*Detroit Lancet.*

Do we want to sue for recognition by the medical profession after that? We think the D. D. S's. can do better.—*S.*

